# High prevalence of childhood multi-drug resistant tuberculosis in Johannesburg, South Africa: a cross sectional study

**DOI:** 10.1186/1471-2334-11-28

**Published:** 2011-01-26

**Authors:** Lee Fairlie, Natalie C Beylis, Gary Reubenson, David P Moore, Shabir A Madhi

**Affiliations:** 1 Faculty of Health Sciences, University of the Witwatersrand, 6 York Street, Parktown, Johannesburg, 2193, South Africa; 2 WHI (Wits Institute for Sexual & Reproductive Health, HIV and Related Diseases), Hospital Street, Chris Hani Baragwanath Hospital, Old Potch Road, Soweto, Johannesburg, 1864, South Africa; 3 National Health Laboratory Service (NHLS), Mycobacteriology Referral Laboratory, Corner de Korte and Hospital Streets, Braamfontein, Johannesburg, 2000, South Africa; 4 Department of Paediatrics Rahima Moosa Mother and Child Hospital, Corner Fuel and Oudshoorn Roads, Newclare, Johannesburg, 2000, South Africa; 5 Department of Paediatrics and Child Health, Metabolic Unit, Chris Hani Baragwanath Hospital, Old Potch Road, Soweto, Johannesburg, 1864, South Africa; 6 Medical Research Council Respiratory and Meningeal Pathogens Research Unit & Department of Science and Technology/National Research Foundation: Vaccine Preventable Diseases; 11th floor Nurses Home, Chris Hani Baragwanath Hospital, Old Potch Road, Soweto, Johannesburg, 1864, South Africa

## Abstract

**Background:**

There are limited data on the prevalence of multi-drug resistant tuberculosis (MDR-TB), estimated at 0.6-6.7%, in African children with tuberculosis. We undertook a retrospective analysis of the prevalence of MDR-TB in children with *Mycobacterium tuberculosis *(MTB) at two hospitals in Johannesburg, South Africa.

**Methods:**

Culture-confirmed cases of MTB in children under 14 years, attending two academic hospitals in Johannesburg, South Africa during 2008 were identified and hospital records of children diagnosed with drug-resistant TB were reviewed, including clinical and radiological outcomes at 6 and 12 months post-diagnosis. Culture of *Mycobacterium tuberculosis *complex (MTB) was performed using the automated liquid broth MGIT™ 960 method. Drug susceptibility testing (DST) was performed using the MGIT™ 960 method for both first and second-line anti-TB drugs.

**Results:**

1317 children were treated for tuberculosis in 2008 between the two hospitals where the study was conducted. Drug susceptibility testing was undertaken in 148 (72.5%) of the 204 children who had culture-confirmed tuberculosis. The prevalence of isoniazid-resistance was 14.2% (n = 21) (95%CI, 9.0-20.9%) and the prevalence of MDR-TB 8.8% (n = 13) (95%CI, 4.8-14.6%). The prevalence of HIV co-infection was 52.1% in children with drug susceptible-TB and 53.9% in children with MDR-TB. Ten (76.9%) of the 13 children with MDR-TB received appropriate treatment and four (30.8%) died at a median of 2.8 months (range 0.1-4.0 months) after the date of tuberculosis investigation.

**Conclusions:**

There is a high prevalence of drug-resistant tuberculosis in children in Johannesburg in a setting with a high prevalence of HIV co-infection, although no association between HIV infection and MDR-TB was found in this study. Routine HIV and drug-susceptibility testing is warranted to optimize the management of childhood tuberculosis in settings such as ours.

## Background

Sub-Saharan Africa has a high burden of human immunodeficiency virus (HIV) and *Mycobacterium tuberculosis *(MTB) infection. Most of the 2.1 million HIV-infected children live in sub-Saharan Africa including 10% in South Africa [1]. An estimated 10% of the 2.9 million new cases of tuberculosis in sub-Saharan Africa during 2007 occurred in children: 38% of all incident tuberculosis cases in sub-Saharan Africa (regardless of age group) were HIV-infected in 2007 [2]. Globally the prevalence of multi-drug resistant tuberculosis (MDR-TB) in adults, defined as resistance to at least isoniazid and rifampicin, was estimated to be 4.6% in 2006 [3]. The prevalence of MDR-TB in South African adults was 1.8% (1.4% - 2.3%) amongst new treatment cases, and 6.7% (5.5% - 8.1%) in previously treated individuals [3].

There are limited data on the prevalence of drug-resistance in African children with tuberculosis. In Cape Town, South Africa, between 2005 and 2007 the prevalence rates of MTB isoniazid resistance were 14.4% and 6.7% of isolates had MDR-TB [4]. These prevalence rates had increased compared to one decade previously when the prevalence of isoniazid resistance was 6.9% (95% CI, 1.1-3.6) and MDR-TB 2.3% (95% CI, 0.9-6.6) [5]. The prevalence of HIV co-infection was 26.6% in children with MDR-TB in the study from Cape Town between 2005 and 2007 [4]. In Bangui, Central Africa Republic, although 15.2% of 165 children with culture-confirmed tuberculosis in whom drug sensitivity testing was available, had MTB strains resistant to at least one drug, only one child (0.6%) had MDR-TB [6]. As children with tuberculosis usually represent cases that have recently acquired infection with MTB, they serve as a crucial indicator of the drug-susceptibility patterns and circulating strain diversity of MTB in a community [5].

The aim of this study was to review the prevalence and clinical outcome of childhood MDR-TB at two hospitals in Johannesburg, South Africa.

## Methods

### Study population and design

The study involved two secondary-tertiary care hospitals, namely Chris Hani-Baragwanath Hospital (CHBH) and Rahima Moosa Mother and Child Hospital (RMMCH) in Johannesburg, South Africa. A retrospective analysis of the laboratory data of children under 14 years of age with microbiologically-confirmed tuberculosis, diagnosed between 1^st ^January 2008 and 31^st ^December 2008, was undertaken.

MTB microscopy, culture and susceptibility test results from children treated at these two hospitals were retrieved electronically from the National Health Laboratory Service (NHLS) Mycobacteriology Referral Laboratory in Johannesburg. DST was not performed routinely on all TB suspects in 2008. Clinicians were guided by the National TB Control Programme and it was at the discretion of the clinician to request additional DST when requesting mycobacterial culture. Thus, DST was only performed in the laboratory at the clinician's request.

Specimens submitted to the Mycobacteriology laboratory for mycobacterial culture with or without additional DST were processed according to WHO guidelines using the N-acetyl-L-cysteine-NaOH decontamination procedure and the Auramine-O technique for direct smear staining. Processed specimens were inoculated into BACTEC™ Mycobacterial Growth Indicator Tube (MGIT™) tubes (Becton-Dickinson, Sparks, Maryland, USA) and incubated at 37°C for 6 weeks in automated MGIT™ instruments. Acid fast bacilli in positive MGIT™ culture tubes were identified as *M. tuberculosis *complex by the HAIN GenoType^® ^Mycobacterium CM assay. For those for whom DST was requested, they would be initially tested against INH and rifampicin using the MGIT™ 960 method. If resistance was found against either or both of these drugs, the isolate would undergo further DST against second line drugs (streptomycin, ethambutol only in the case of INH mono-resistance, or streptomycin, ethambutol, ethionamide, kanamycin and ofloxacin in the case of rifampicin mono-resistant MTB or MDR-TB) using the MGIT™ 960 method. DST against pyrazinamide was performed only if requested by the attending clinician. Blood specimens were inoculated at the bedside into BACTEC™ Myco/F Lytic medium and these bottles were incubated for 6 weeks in automated BACTEC™ 9240 incubators in the laboratory. Identification of MTB or M.avium complex from positive for AFB-blood cultures was by nucleic acid probe (Accuprobe, Genprobe). DST on MTB -positive blood cultures was also by the MGIT™ 960 method after subcultirung the isolate from blood into a MGIT™ tube.

MTB susceptibility was categorized as susceptible based on isoniazid and rifampicin susceptibility (no further DST testing against other drugs was performed in these cases); isoniazid mono-resistance if resistant to isoniazid only; rifampicin mono-resistance if resistant to rifampicin only; MDR-TB when resistance to both isoniazid and rifampicin was present; and extensively drug-resistant (XDR-TB) when resistance to isoniazid, rifampicin, a fluoroquinolone and kanamycin was present.

Children diagnosed with MDR-TB at either of the hospitals were referred to Sizwe Hospital, a dedicated MDR-TB treatment facility in Johannesburg. The standard of care for HIV-infected children at the study sites during 2008 included highly active antiretroviral treatment (HAART) and co-trimoxazole prophylaxis based upon South African Department of Health guidelines [7]. Additionally, the standard of care for children with MDR-TB admitted to Sizwe Hospital included treatment with pyrazinamide, ethionamide, ethambutol, amikacin and ofloxacin in the intensive phase of treatment with the addition of terizidone and/or kanamycin if additional resistance patterns were present. Continuation phase MDR-TB treatment consisted of ethionamide, ethambutol and ofloxacin [8].

HIV status was ascertained by searching the laboratory database for HIV DNA PCR results in children <18 months, or HIV ELISA results in children ≥18 months of age. Children were recorded as being HIV-infected, HIV-uninfected or HIV unknown. CD4+ counts and HIV viral load by PCR quantification, undertaken within three months of the date when the child was investigated for tuberculosis, were extracted for HIV-infected children. 'Baseline' was defined as the date when positive samples had been submitted for investigation of tuberculosis.

A clinical-record review was performed for children who had drug-resistant MTB, and the following data recorded: baseline weight-for-age z-scores; clinical data indicative of symptomatic tuberculosis at the time of submission of the specimen from which MTB was cultured; antituberculosis treatment history; tuberculosis contact history; outcome (responding well to treatment, died, or lost to follow-up). A 3-month period either side of baseline was allowed for the collection of clinical data. Additionally, for HIV-infected children, World Health Organization (WHO) clinical stage and details of HAART as well as response to HAART were recorded. Time from tuberculosis diagnosis and initiation of anti-tuberculosis treatment was calculated based on subject record review or information received from the City of Johannesburg Tuberculosis Program. Six- and 12-month clinical and radiological data were recorded where available.

### Statistical analysis

The overall prevalence of drug-resistant MTB for case isolates (number of children with culture-confirmed tuberculosis) in which MTB complex was cultured and used as a denominator was calculated using the chi square test with exact binomial 95% confidence intervals (95%CI), or Fisher's exact test, as appropriate. Prevalence of drug-resistant TB was also calculated using only TB episodes (number of children with drug-resistant tuberculosis) in which drug resistance testing was undertaken as a denominator. Statistical analyses were performed using STATA version 11.0 software (StataCorp, College Station, Texas, USA). All tests were conducted at a significance level of 0.05.

### Ethics approval

Ethics approval was granted by the Human Research Ethics Committee (Medical) of the University of the Witwatersrand. A waiver for informed consent on the part of identified subjects was granted by the Ethics Committee in accordance with this being a retrospective review.

## Results

### Drug susceptibility testing

In 2008, 1317 children were treated for tuberculosis between the two hospitals based on information accessed through an electronic register which is maintained at both facilities' TB Focal Points.

**Figure 1:**
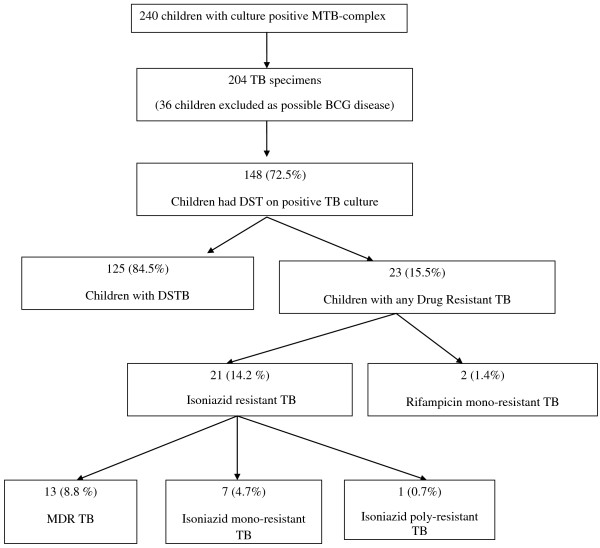
Children with culture positive TB- drug susceptibility patterns

MTB complex were cultured from 240 children in 2008, including 36 (15.0%) isolates that were considered to be possible *Mycobacterium bovis *bacillus Calmette-Guérin (BCG) strain, based on being exclusively identified from lymph node aspirates. Of these specimens, seven demonstrated drug- resistant TB complex, all from right axillary lymphadenitis (1 MDR-TB, 5 INH mono-resistance and 1 INH poly-resistance (INH and pyrazinamide). Pyrazinamide resistance patterns were only available for the one lymph node isolate which demonstrated pyrazinamide resistance.MTB speciation was only undertaken (on clinician request) in one of these 36 isolates, which was confirmed as *M. bovis-*BCG. As it was not possible to ascertain retrospectively whether isolates obtained from lymph node aspirates were BCG in all cases, children in whom MTB complex was only identified from lymph node aspirate samples were excluded from further analysis as bias may have been introduced by including these cases: ages were not available for all children, and the location of lymph node aspiration was not available for drug-sensitive cases. (Figure 1). Two hundred and four children had culture-confirmed tuberculosis; sites of identification of the isolates from these 204 children included gastric aspirates (n = 95; 46.6%), sputum (n = 75; 36.8%), blood (n = 16; 7.8%), cerebrospinal fluid (n = 5; 2.5%), tracheal aspirate (n = 5; 2.5%), pleural fluid (n = 4; 2.0%), bone marrow (n = 1; 0.5%), synovial fluid (n = 1; 0.5%), tissue (n = 1; 0.5%) and urine (n = 1; 0.5%). Only one positive specimen was included per child and all duplicates were removed from analysis. Drug susceptibility testing, at the discretion of the attending clinician was conducted on samples from 148 (72.5%) of the 204 children.

### HIV prevalence in the cohort

Ninety- nine (48.5%; 95% CI, 41.5 to 55.6%) of the 204 children with culture-confirmed tuberculosis were HIV-infected, 77 (37.7%; 95% CI, 31.1 to 44.8%) were HIV-uninfected and the HIV infection status was unknown in the remaining 28 (13.7%; 95% CI, 9.3 to 19.2%) children. Baseline immunological results were available for 91 (91.9%) of the HIV-infected children (Table 1). The median absolute CD4+ cell count was 312 cells/μl (Interquartile Range [IQR], 113 to 743) and the median CD4+ percentage, 17.5% (IQR, 8.5 to 23.2%). Baseline viral loads were available for 87 (87.9%) HIV-infected children, with a median of 4.60 log_10 _viral copies/ml (IQR, 1.98 - 5.28). The CD4+ cell count and HIV viral load of the HIV-infected population did not differ with respect to those in whom drug susceptibility testing was undertaken. Information regarding access to HAART was limited as our data were laboratory-based and folder reviews were only conducted for those children with drug-resistant tuberculosis. Of the seven HIV-infected children with MDR-TB, six were on HAART, and three of these had failed first-line HAART regimens, and were being treated with second line HAART regimens.

**Table 1:** Comparison of drug susceptible and drug resistant cases at baseline

	Drug susceptible TB	MDR TB	Rifampicin mono-resistance	INH Resistance with no Rifampicin resistance
Number of children with positive cultures submitted for DST	125	13	2	8

HIV infected	64 (51.2%)	7 (53.8%)	1 (50.0%)	6 (75.0%)

HIV uninfected	42 (33.6%)	6 (46.2%)	1 (50.0%)	2 (25.0%)

HIV unknown	19 (15.2%)	0 (0.0%)	0 (0.0%)	0 (0.0%)

Baseline CD4+ count	351 (126 - 825)	180 (36 - 264)	107	467 (246 - 831)
(median, IQR)				

Baseline CD4 percentage (median, IQR)	17.2 (8.2 - 23.0)	14.2 (2.2 - 23.2)	5.3	28.2 (14.6 - 40.4)

Baseline log HIV Viral load (median, IQR)	4.49 (2.34 - 5.26)	5.19 (4.11 - 5.52)	4.67	3.17 (1.40 - 4.66)

Age months	24 (12 - 72)	25 (5 - 84)	78 (48 - 108)	60 (7 - 114)
(median, IQR)				

Weight for age z-score	Not available	-2.8 (-3.5- -0.5)	-2.2 (-3.1- -1.3)	-2.0(-4.8- -0.2)
(median, range)				

### Drug susceptibility testing

Among the 148 isolates in which drug susceptibility testing was undertaken, 23 (15.5%: 95% CI, 10.1 - 22.4%) exhibited non-susceptibility to at least one first-line anti-tuberculosis drug. The overall prevalence of isoniazid resistance was 14.2% (95% CI, 9.0 to 20.9%; n = 21) and rifampicin-resistance was 10.1% (95% CI, 5.8 to 16.2%; n = 15). Isoniazid mono-resistance was prevalent in seven (4.7%; 95% CI, 1.9 to 9.5%) cases and the isolate from one (0.7%; 95% CI, 0.02 to 3.7%) child had isoniazid and ethambutol poly-resistance. In addition, two (1.4%; 95% CI, 0.2 - 4.8%) cases were associated with rifampicin mono-resistance. The overall prevalence of MDR-TB (n = 13) was 8.8% (95% CI, 4.8 to 14.6%) in isolates in which drug susceptibility testing was undertaken.

### MDR-TB episodes

There was no difference in the prevalence of HIV between children with drug-susceptible tuberculosis and those with MDR-TB (p = 0.771) (Table 1). Additionally, there were no significant differences in age, CD4+ percentage, absolute CD4 count, or HIV log_10 _viral load between HIV-infected children with MDR-TB compared to those with drug-susceptible tuberculosis. The 13 children with MDR-TB included eight children with concurrent streptomycin resistance, four with concurrent ethambutol resistance and one child with ethionamide resistance (Table 2). Only four (30.8%) of the 13 children with MDR-TB were known to have been in contact with adult tuberculosis cases. The median age of children with positive TB contacts was 60.9 months (range 18.5-144 months). None of these adult contacts were admitted to the MDR-TB treatment facility or were known to have had drug susceptibility testing done on their MTB isolates. One of the contacts of a child with MDR-TB was known to have defaulted anti-tuberculosis treatment, and the known contact of another had demised.

**Table 2:** Baseline characteristics of children with MDR TB

	Age (m)	Sex	TB previous	TB contact	Baseline Clinical	HIV	ART at baseline (m)	CD4 # baseline	CD4% baseline	RNA PCR baseline	Time to appropriate treatment (m)	WAZ-score	Drug Resistance
1	2	F	No	No	αExprem, CLD	Neg					LTFU^$^	-3.1	MDR

2	3.4	M	No	No	ααExprem, CDH,CLD, ASD	Neg					Died 3 days after investigation	-3.9	MDR

3	3.8	M	No	No	Well	Neg					4.0	-0.5	MDR, Strep

4	5.2	M	No	No	WHO IV Marasmus	Pos	2				2.0	-1.8	MDR

5	11.9	M	No	No	Kwashiorkor	Neg					3.3	-3.0	MDR

6	18.5	M	No	Yes mom defaulted	Kwashiorkor	Neg					4.5	-2.0	MDR, Strep

7	25	M	No	Yes (father)	WHO IV Kwashiorkor	Pos	No (Defaulted)	264	23.2	4.9	15	-2.3	MDR, Strep

8	29.2	F	No	Unk	UWFA	Neg					3	-3.2	MDR Strep

9	62	F	no	no	WHO IV	Pos	1	381	23.2	6.3	0.8	-3.5	MDR

10	83.7	F	Yes 2001 and 2002 and 2008 ∞	U	WHO IV CLD	Pos	1^st ^line 23 m. 2nd line regimen 4 m. Poor adherence	105	5.1	1.4	0.3	-3.5	MDR, EMB, Strep

11	96.7	F	No	Yes	WHO IV	Pos	Yes 36 m Defaulter	9	1.1	5.1	1.3	-2.7	MDR, Strep, EMB

12	144	F	Yes 2001, 2005, 2007 ^	Yes mom demised from TB 2004	WHO III	Pos	11 m 1^st ^line Poor adherence 2nd line regimen 15 months	254	32	4.1	2.5	-1.0	MDR, EMB, Strep

13	144	M	No	U	WHO IV	Pos	2	36	2.2	5.5	1.5	-2.4	MDR, Strep, Eth, EMB

Median	24							180	14.2	5.19	2.5	-2.8	

α Ex-premature infant, CLD= chronic lung disease.

αα CHD=Congenital diaphragmatic hernia, ASD=Atrial septum defect.

^$ ^LTFU= Lost to follow up

^ Resistant to ethambutol and isoniazid: treated with ethionamide and ciprofloxacin.

∞ Admitted to MDR-TB Hospital

### Management of children with MDR-TB

Two (15.4%) children with MDR-TB received no antituberculosis treatment, including one child who died and one child who defaulted follow-up. A third child who received two months of empiric therapy for drug susceptible tuberculosis, and then defaulted care was recalled and re-investigated by attending physicians 15 months after the initial investigation revealed MDR-TB. The repeat MTB cultures in this child were negative and based on the child being clinically well with a normal chest radiograph, the attending physician opted not to initiate MDR-TB treatment.

Ten (76.9%) of the 13 children with MDR-TB received appropriate anti-tuberculosis treatment. The median time to tracing and initiation of appropriate anti-tuberculosis treatment from time of specimen submission in these children was 2.5 months (range 0.3-15 months). Seven children (53.8%) with MDR-TB and one child with rifampicin-resistant tuberculosis were started on first-line antituberculosis treatment initially. Two of the 13 children were started on MDR-TB treatment within one month of specimen submission, both of whom were diagnosed with MDR-TB based on the line probe assay.

Seven children were admitted to the MDR-TB treatment facility for further management for a median time period of 6.0 months (range 3 - 17 months). Three children were treated at the study-hospitals with MDR-TB treatment regimens as they were too unstable for referral to the MDR treatment-facility.

### Outcome of children with MDR-TB

Four children (30.8%) with MDR-TB died at a median of 2.8 months (range 0.1-4.0 months) from the time when the initial sample identifying MDR-TB was submitted. This included three HIV-infected children on HAART who had been initiated on MDR-TB treatment at a median time of 1.5 months after sample submission for mycobacterial culture. These three children were treated in the study-hospitals as they were too unstable for down-referral to the MDR-TB treatment facility. The fourth child, an HIV-uninfected two-month old, was an ex-premature infant with an atrial septal defect, congenital diaphragmatic hernia and chronic lung disease. This child died within three days of specimen submission and was not initiated on anti-tuberculosis treatment. One of the children who died had MDR-TB meningitis and the other three children died from severe pneumonia. There was no correlation between weight for age z-scores and death.

At 12-month follow-up (table 3) the mean weight-for-age z-score was -1.6 (range -3.1 to 0.4) in the nine survivors. In three HIV-infected children in whom follow-up results were available, the median CD4+ count was 291 cells/μl (range 285 - 2050 cells/μl) with a median CD4+ percentage of 16.4% (range 11.1 - 33.0%). The median HIV viral load was 4.7 log_10 _copies/ml (range 1.4 - 5.0). One child had worsening changes on chest radiograph. All other children with severe chest radiographic changes at baseline were noted to have improved by 12 months. All children who had been admitted to the MDR-TB treatment facility were retained in care, with none defaulting treatment follow-up. There were no deaths between the 6 and 12-month follow up periods.

**Table 3:** 12 month outcomes of children with MDR TB

					Baseline			12-month follow-up			
				
	Age (m)	Sex	HIV	Outcomes	WAZ-score	CD4 #	CD4%	WAZ-score	CD4 #	CD4%	RNA
1	2	F	Neg	Lost to follow-up	-3.1						

2	3.4	M	Neg	Died 0.1 m	-3.9						

3	3.8	M	Neg	*MTBH 3 m	-0.5			-1.6			

4	5.2	M	Pos	Died 4.0 m	-1.8						

5	11.9	M	Neg	MTBH 6 m	-3			-0.4			

6	18.5	M	Neg	MTBH 6 m	-2			-0.9			

				Traced							
7	25	M	Pos	Re-investigated	-2.3	264	23.2	-3.1	297	21.7	5

8	29.2	F	Neg	MTBH 6 m	-3.2			-2			

9	62	F	Pos	MTBH 7 m	-3.5	381	23.2	-0.9			

10	83.7	F	Pos	MTBH 17 m	-3.5	105	5.1	-2.1	285	11.1	4.7

11	96.7	F	Pos	Died 1.8 m	-2.7	9	1.1				

12	144	F	Pos	MTBH 12 m	-1	254	32	-1.6	513	14.4	2.9

13	144	M	Pos	Died 4.0 m	-2.4	36	2.2				

*MTBH= MDR TB treatment facility

## Discussion

This study demonstrates a high prevalence of MDR-TB in children in Johannesburg, South Africa. MDR-TB prevalence of 8.8% in cases on which drug-susceptibility testing was performed in our study are higher than the point-prevalence of MDR described in other African paediatric studies (2.3-6.7%) [4-6]. Drug resistant-tuberculosis was prevalent in 16% of children with culture-confirmed tuberculosis, whose specimens underwent susceptibility testing. Notably, only four of the children with MDR-TB had a known exposure to an adult tuberculosis contact, none of whom were diagnosed with MDR-TB. Contact tracing in our context is poorly performed; a study at three Johannesburg academic hospitals (including CHBH and RMMCH) showed that 15% of 113 severely malnourished paediatric inpatients had contact with an adult recently diagnosed with tuberculosis, and none were offered IPT [9]. Lack of MDR-TB household contacts in our cohort suggests transmission outside the home, but further epidemiologic patterns could not be ascertained. As childhood tuberculosis is a marker of ongoing adult disease and MTB transmission, our study suggests that MDR-TB transmission occurred within the community as well as within the child's household [10-12]. As childhood tuberculosis also serves as a measure of the drug-susceptibility patterns of MTB being transmitted within a community, our study indicates that there is a high prevalence of MDR-TB circulating in our setting.

High rates of drug-resistant MTB may impact on tuberculosis preventive therapy strategies that have recently been recommended in the South African national TB guidelines (2009) [13]. In children with INH mono-resistance or poly-resistance, without rifampicin resistance, 75% were HIV infected. The high prevalence of isoniazid resistance (14.2%; 95% CI, 9.0 to 20.9%) demonstrated in our study raises questions about the effectiveness of isoniazid preventive therapy in our community [14]. The high prevalence of isoniazid resistance in this setting may also, in part, explain the lack of efficacy of primary isoniazid prophylaxis in improving tuberculosis-free survival in HIV-infected children in our setting [15].

Current South African tuberculosis guidelines (2009) [13] recommend tuberculosis microscopy and culture in adult tuberculosis suspects with smear-negative disease who are ill and not responding to antibiotics, regardless of HIV status. No drug susceptibility testing is recommended for smear-positive adult cases. One specimen for MTB culture and sensitivity is sent for high-risk TB suspects [13]. In child tuberculosis suspects, smear and culture of sputum or gastric aspirates is recommended, and paediatric studies have demonstrated that it is possible to perform these investigations in many settings, in children of varying ages; however they are often not done routinely in outpatient settings [16,17]. Currently, drug susceptibility testing is not routinely performed on MTB isolates cultured from children and drug susceptibility testing of isolates in children with tuberculosis in our cohort was performed at clinician discretion in an academic hospital setting, deviating from current local guidelines [13]. Line probe assays have been demonstrated to provide drug susceptibility results within 5 - 7 days in high-throughput laboratories in microscopy-positive specimens, or once MTB is cultured; [18] this test still requires evaluation in specimens derived from children. Future plans nationally in South Africa are to enhance the capacity for routine testing of all smear-positive adult and paediatric specimens using this assay, in order to report drug sensitivity patterns more rapidly.

High MDR-TB attributable mortality (30.8%) was evident in our cohort at a median of 2.8 months after investigation. Of the children who demised, three were HIV-infected on HAART and the fourth child was an HIV-uninfected ex-premature infant with associated congenital abnormalities. The three children who demised and were started on MDR treatment, started MDR-TB therapy at a median of 1.5 months after specimen submission, demonstrating no significant delay in access to specific therapy compared to the survivors. This mortality is higher than described in a previous study from the Western Cape where 10% of children with MDR-TB died over a four year study period, 50% of whom were HIV-infected; [19] and compared to 3.5% described in children with culture-confirmed MDR-TB receiving community based treatment in Lima, Peru [20]. In our study, one child died of MDR-TB meningitis and the other three children died of pneumonia and acute respiratory failure. All children died in hospital as they were too unstable to be transferred to a MDR-TB treatment facility. Appropriate isolation facilities are not widely available in many general hospital settings in South Africa, and therefore these children constituted an infection control dilemma when known to have MDR-TB, as well as prior to their MDR-TB diagnoses. Even if the diagnosis of MDR-TB is expedited through modern diagnostic techniques, increased risk of nosocomial MDR-TB transmission to fellow patients and healthcare workers is likely if adequate isolation facilities are not available [21]. With increased MDR-TB rates evident especially in high HIV prevalence areas, infection control needs to be addressed urgently.

Our study has limitations, including the fact that we may have overestimated the true MDR-TB prevalence in children in Johannesburg, as patients attending referral hospitals may be at higher risk for drug-resistant tuberculosis compared to those investigated and treated at primary care facilities. In the Western Cape (2003 - 2005), however, there was no difference in drug susceptibility patterns between MTB isolates from hospitalized compared to community-based childhood TB cases [5]. Furthermore, in our study, DST was performed in only 72.5% of children with culture-confirmed tuberculosis at clinician discretion, which may have introduced some bias. Our analysis also excluded all specimens with culture-confirmed mycobacterial isolates derived from lymph node aspirates, in order to control for the possibility of drug resistance being associated with culture of *M. bovis-*BCG [22,23] which is innately resistant to pyrazinamide and variably resistant to isoniazid [24-26]. Drug susceptibility testing against pyrazinamide is not routinely performed in our setting and is only performed on clinician request. As we only had pyrazinamide resistance patterns for one specimen, we could not make any assumptions about whether these lymph node isolates were *M.bovis *or *M.tuberculosis *based on pyrazinamide resistance. As the study was retrospective and record-based, contact history data may be inaccurate. Detailed data on children with drug-susceptible MTB was not available for this study, as information was collected from laboratory records.

This study reports MTB and MDR-TB/HIV co- infection rates in the HAART era. In our cohort there were high rates of HIV co-infection. There was, however, no difference in HIV co-infection prevalence between children diagnosed with drug-susceptible and MDR-TB (P = 0.771), suggesting that HIV infection does not appear to be a risk factor for MDR-TB in this setting. There was also no significant difference between absolute CD4+ count, CD4+ percentage and HIV viral load in drug-susceptible and MDR-TB cases. Our study findings, nevertheless, highlight the importance of ascertaining the HIV-infection status of every child diagnosed with tuberculosis.

## Conclusion

In conclusion, we have demonstrated a high prevalence of drug-resistant MTB in a cohort of children diagnosed with culture-confirmed tuberculosis in Johannesburg, South Africa; this likely represents a large burden of undiagnosed drug-resistant MTB in household and community adult contacts of these children. All child tuberculosis suspects in settings with a high prevalence of tuberculosis and HIV should have confirmation of their HIV infection status. Furthermore, we recommend that routine DST should be performed on MTB isolates obtained from children with culture-confirmed TB in these high-burdened settings.

## Competing interests

The authors declare that they have no competing interests.

## Authors' contributions

LF and SAM conceived of the study. LF designed the study, collected data, drafted the manuscript and coordinated the revisions to the manuscript.NCB collected data and participated in study design and drafting of manuscript.DPM performed the statistical analysis and contributed to drafting of the manuscript.GR collected data and contributed to drafting of the manuscript. All authors read and approved the final manuscript

## Pre-publication history

The pre-publication history for this paper can be accessed here:

http://www.biomedcentral.com/1471-2334/11/28/prepub
